# Preventing Lower Limb Graft Thrombosis after Infrainguinal Arterial Bypass Surgery with Antithrombotic Agents (PATENT Study): An International Expert Based Delphi Consensus

**DOI:** 10.3390/jcm12093223

**Published:** 2023-04-30

**Authors:** Lorenz Meuli, Thomas Stadlbauer, Barbara E. Stähli, Christine Espinola-Klein, Alexander Zimmermann, on behalf of the PATENT Study Collaborators

**Affiliations:** 1 Department of Vascular Surgery, University Hospital Zürich, University of Zürich, 8091 Zürich, Switzerland; 2 Department of Cardiology, University Heart Centre, University Hospital Zürich, University of Zürich, 8091 Zürich, Switzerland; 3 Centre for Cardiology, Cardiology III-Angiology, University Medical Centre of the Johannes Gutenberg University of Mainz, 55131 Mainz, Germany

**Keywords:** antithrombotic therapy, peripheral arterial disease, direct factor Xa inhibitor, bypass surgery, antiplatelet therapy

## Abstract

(1) Background: High-level evidence on antithrombotic therapy after infrainguinal arterial bypass surgery in specific clinical scenarios is lacking. (2) Methods: A modified Delphi procedure was used to develop consensus statements. Experts voted on antithrombotic treatment regimens for three types of infrainguinal arterial bypass procedures: above-the-knee popliteal artery; below-the-knee popliteal artery; and distal, using vein, prosthetic, or biological grafts. The treatment regimens for these nine procedures were then voted on in three clinical scenarios: isolated PAOD, atrial fibrillation, and recent coronary intervention. (3) Results: The survey was conducted with 28 experts from 15 European countries, resulting in consensus statements on 25/27 scenarios. Experts recommended single antiplatelet therapy after above-the-knee popliteal artery bypasses regardless of the graft material used. For below-the-knee popliteal artery bypasses, experts suggested combining single antiplatelet therapy with low-dose rivaroxaban if the graft material used was autologous or biological. They did not recommend switching to triple therapy for patients on oral anticoagulants for atrial fibrillation or dual antiplatelet therapy in any scenario. (4) Conclusions: Great inconsistency in the antithrombotic therapy administered was found in this study. This consensus offers guidance for scenarios that are not covered in the current ESVS guidelines but must be interpreted within its limitations.

## 1. Introduction

The endovascular revolution has narrowed the indications for bypass surgery for many patients with peripheral arterial occlusive disease (PAOD). However, bypass surgery remains the best procedure for cases with complex lesions and a vein suitable for reconstruction [1]. Despite that, the evidence on antithrombotic therapy after bypass surgery is still limited.

Antiplatelet Trialists’ Collaboration showed in a meta-analysis from 1994 that single antiplatelet therapy (SAPT) is associated with a 43% reduction in the relative risk of bypass occlusion. Since then, few trials have been conducted on assessing different antithrombotic regimens and their efficacy in preventing occlusions in different clinical scenarios [2]. The “Dutch Bypass Oral Anticoagulant or Aspirin Study” (BOA) reported that therapeutic anticoagulation with vitamin K antagonists given orally demonstrated a reduction in occlusion rates for venous bypass grafts but not for prosthetic bypass grafts [3]. However, these results were found in a subgroup analysis whereas the overall trial did not find significant differences between the treatment groups. Furthermore, the risk of fatal and intracranial bleeding was significantly increased in the vitamin K group. Another trial, “Clopidogrel and Acetylsalicylic Acid in Bypass Surgery for Peripheral Arterial Disease” (CASPAR), investigated the outcomes of antiplatelet therapy. That trial showed no difference in graft patency and amputation-free survival using dual antiplatelet therapy (DAPT) with acetylsalicylic acid (ASA) and clopidogrel compared with ASA and placebo. In a subgroup analysis for prosthetic below-the-knee bypass grafts, DAPT led to superior results compared to monotherapy with ASA [4].

More recently, the “Vascular Outcomes Study of Acetylsalicylic Acid along with Rivaroxaban in Endovascular or Surgical Limb Revascularisation for Peripheral Artery Disease” (VOYAGER PAD) demonstrated the benefits of using ASA in combination with low-dose rivaroxaban (dual pathway inhibition: DPI). It showed the benefits on a large albeit heterogeneous population after successful infrainguinal revascularisation [5]. It is worth noting that the study included more than 6564 patients of which only 1448 (22%) have undergone bypass surgery. This evidence formed the basis for the recommendations on antithrombotic therapy for PAOD published recently in a consensus document from the European Society of Cardiology and found its way into the Canadian national guidelines for the first time in 2022 [6,7]. However, the data on antithrombotic treatment of patients undergoing surgical revascularisation for chronic limb ischaemia (CLTI) is still very heterogeneous [8]. On top of that, about 15% of patients with PAOD are already on anticoagulant therapy due to other cardiovascular conditions [9].

This study, therefore, aims to establish consensus recommendations for antithrombotic therapy after infrainguinal bypass surgery for PAOD.

## 2. Materials and Methods

Expert panel: Inclusion criteria for invited experts were: (1) ≥10 years of experience in managing patients with PAOD, (2) an institutional caseload of ≥20 peripheral bypass procedures per year, (3) personal scientific interest in the antithrombotic management of patients with PAOD and peripheral bypass surgery, and (4) affiliation to a European vascular centre. Potentially eligible vascular specialists were identified through screening the author lists of relevant publications, e.g., European Society for Vascular Surgery (ESVS) Guidelines, and obtained from the ESVS country representatives list. Experts were reached out through email with the study protocol and the anticipated time commitment. Eligibility was assessed during Delphi round 1 based on self-reporting of the following: indication of affiliation, declaration of annual caseload, and confirmation of scientific expertise in the field.

Definitions of bypass surgery: Infrainguinal bypass surgeries were grouped into three groups anatomically (1–3) based on the extent of the bypass and were grouped again into another three groups (a–c) based on the graft material of the used conduit. All combinations, i.e., 1a to 3c, were presented to the experts:

1. Above-the-knee popliteal artery: this is defined as a bypass from the common femoral artery (CFA) or distal to the popliteal artery (P1 segment or P2 segment).
2. Below-the-knee popliteal artery: this is defined as a bypass from the CFA or distal to the popliteal artery (P3 segment) or tibioperoneal trunk.
3. Distal: this is defined as a bypass from the CFA or distal to the isolated anterior or posterior tibial or peroneal artery or distal of them.

(a) Autologous: this is defined as purely autologous venous bypass including upper limb veins.
(b) Prosthetic: this is defined as synthetic grafts with or without drug coating, including any composite bypass with vein and prosthetic material.
(c) Biological: this includes xenografts (e.g., bovine, ovine, or porcine) and homografts.

Definitions for antithrombotic therapy: Antiplatelet therapy (APT) included ASA, clopidogrel, ticagrelor, and prasugrel. DAPT was defined as any combination of two antiplatelet drugs. Oral anticoagulation (OAC) included vitamin K oral antagonists and direct factor Xa or thrombin inhibitors (direct oral anticoagulants, DOACs). For DOACs, low-dose vs full-dose was specified.

**Figure 1:**
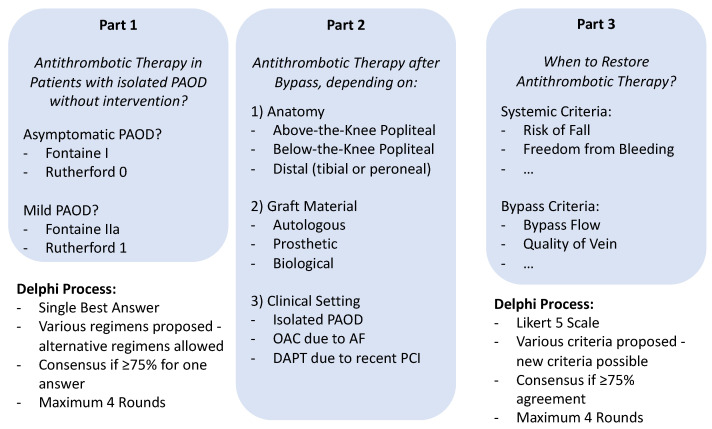
Delphi Process. The Delphi process was divided into three parts and contained a maximum of four rounds. PAOD = peripheral arterial occlusive disease; OAC = oral anticoagulation; AF = atrial fibrillation; DAPT = dual antiplatelet therapy; PCI = percutaneous coronary intervention.

Delphi survey: We used a modified Delphi process using SurveyMonkey (Palo Alto, CA, USA). After agreeing to participate, experts were provided with access to each survey round via a secure institutional email. Consensus definitions and the Delphi process were prespecified in a published protocol study available at https://osf.io/tn6km (accessed on 28th April 2023) and provided in the Supplementary Material. Figure 1 shows the Delphi process. The survey included three main parts:

- First part: experts were asked to provide general recommendations for antithrombotic therapy in patients with asymptomatic PAOD and patients with mild intermittent claudication not treated with bypass surgery, The possible answers included: No antithrombotic therapy, ASA, clopidogrel, DPI, full dose OAC, or other (open-ended option).
- Second part: experts were asked to recommend an antithrombotic regimen for each of the nine distinct combinations of bypass surgery introduced earlier in three clinical scenarios:
  (a) A patient with isolated PAOD with no other medical condition that requires antithrombotic therapy.
  (b) A patient with atrial fibrillation with a CHA2DS2-VASc score of ≥ 3 on OAC [10].
  (c) A patient on DAPT due to a recent (<6 months) percutaneous coronary intervention (PCI) for acute or chronic coronary syndrome.

The possible answers included: No antithrombotic therapy, SAPT, DAPT, OAC (low-dose or full-dose), SAPT in combination with OAC (low-dose or full-dose), and other (open-ended option). After consensus, the treatment duration for each scenario was evaluated.

- Third part: experts were asked to vote on a 5-point Likert scale (1 = strongly disagree, 2 = disagree, 3 = neither agree nor disagree, 4 = agree, 5 = strongly agree) on a set of potential decision criteria to restore the antithrombotic treatment regimen to the preoperative state. The experts were asked to add further criteria during the Delphi process.

In Delphi round 1, additional basic information on the institutional experience and the preferred graft material for each bypass group were gathered.

Consensus definition: For clinical scenarios, consensus is achieved if ≥75% of the experts proposed the same treatment strategy. For decision criteria, consensus is achieved if ≥75% of the experts voted concordant on the 5-point Likert scale, i.e., disagreed (1 or 2) or agreed (4 or 5).

After completing each Delphi round, the next questionnaire was adopted according to the results of the previous round. For clinical scenarios where consensus was not achieved, treatment regimens with less than the ratio of votes expected, i.e., <25% in four different responses, were excluded. In round 2, the option “OAC (low dose or full dose)” was divided into two separate answers (one for low-dose OAC and one for full-dose OAC) if the answers received the necessary percentage of votes in round 1.

From Delphi round 2 on, the percentage of votes for each antithrombotic regimen and the percentage of concordance of the previous rounds were visible to enable the experts to revote considering the previous results from other experts. If no consensus was reached in round 3, the Delphi process was halted for decision criteria questions, and the results were summarized and presented as a lack of consensus.

After consensus was reached for clinical scenarios, experts were asked to suggest a minimum treatment duration for the proposed treatment regimen. For clinical scenarios without a consensus after three rounds, a statement was formulated with the top two most frequently chosen answers if applicable. For this statement, treatment duration was asked among the experts who agreed with it.

Data Collection: Experts were given four weeks to complete each round. Three reminders were sent, and the round was closed after a maximum of six weeks. Data collection took place from November 2021 to May 2022.

Data were summarised as the percentage of agreement, and in which round the consensus was achieved (R1–R4). To explore any differences among experts completing the entire survey and experts who discontinued participation after round 1, the self-reported institutional caseload was compared using the Mann-Whitney U Test. The institutional caseload was summarised as a median and quartiles (interquartile range (IQR): Q1 to Q3) using R Studio version 3.6.3 for macOS. The results of the Delphi study were compared against the current ESVS guidelines and the relevant randomised controlled literature in the field [3,4,5,11,12].

## 3. Results

**Figure 2:**
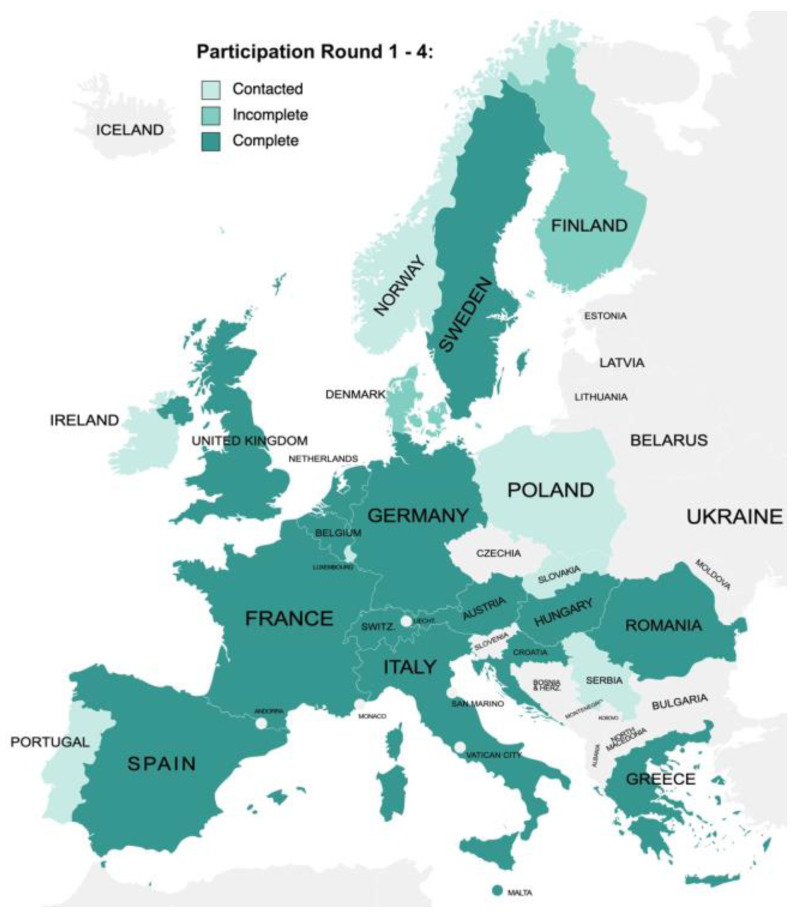
Residence of the expert panel. 95 experts from 24 European countries were contacted, of which 43 agreed to participate. Of the experts contacted, 28 from 15 European countries completed all rounds of the survey.

Ninety-five experts were invited, of whom 43 agreed to participate. Twenty-eight experts from 15 European countries responded to all rounds of the survey (a response rate of 65%) (Figure 2). All experts were vascular specialists affiliated with a European vascular centre (25 vascular surgeons, 2 angiologists, 1 interventional cardiologist). Experts have a median annual institutional caseload of 26 (IQR: 19 to 52) above-the-knee popliteal artery, 38 (24 to 61) below-the-knee popliteal artery, and 20 (10 to 24) distal bypass procedures. Participants not completing all rounds of the survey (*n* = 15) were working at equally respectable centres to those whom completed all four rounds and did not disagree with the content or the format of the survey. No statistically significant difference in the annual institutional caseloads was found between non-completing participants and completing participants. All participants met the eligibility criteria using the data self-reported in round 1. With any anatomical group, autologous veins were the preferred conduits with 75% for above-the-knee popliteal artery, 93% for below-the-knee popliteal artery, and 100% for distal bypass procedures. Details of the expert panel and preferred graft material are shown in Supplementary Table S1.

### 3.1. General Recommendations

For the first part of the Delphi survey, consensus was achieved after two rounds for both clinical scenarios (Table 1). For patients with asymptomatic PAOD (Fontaine stage I, Rutherford grade 0), experts recommended treatment with ASA (76% agreement). For patients with mild PAOD (Fontaine stage IIa, Rutherford grade 1), the experts also recommended treatment with ASA (77% agreement).

### 3.2. Antithrombotic Therapy after Bypass Surgery

After round 4, consensus on treatment recommendations was achieved in 25 of the 27 different proposed clinical scenarios (Table 2, Table 3 and Table 4).

#### 3.2.1. No Previous Antithrombotic Therapy

For above-the-knee popliteal artery bypasses in patients with no antithrombotic therapy prior to bypass surgery, experts recommended SAPT for all used graft materials (Table 2). For below-the-knee popliteal artery bypasses, experts recommended consideration of 6 months or permanent DPI and/or permanent SAPT if the used graft material was autologous (93%) or biological (85%). No consensus was achieved on patients with prosthetic below-the-knee popliteal artery bypass grafts. In that scenario, most of the experts recommended DAPT (56%), and the remainder (44%) recommended DPI. For autologous distal bypass graft, experts recommended permanent SAPT -DPI for 12 months or permanent should be considered (76%). For biological distal bypass graft permanent SAPT, additional OAC for 6 months or permanent DPI should be considered (76%). No consensus was achieved on patients with prosthetic distal bypass grafts. In that scenario, most of the experts recommended DPI (67%), and the remainder (33%) recommended SAPT with OAC.

#### 3.2.2. Oral Anticoagulation Due to Atrial Fibrillation

For patients on OAC due to atrial fibrillation (Table 3), experts recommended that OAC should be continued after discharge regardless of the bypass procedure performed. If those patients had a distal prosthetic bypass, additional treatment with SAPT was recommended. If they had below-the-knee popliteal artery prosthetic or biological, or distal autologous or biological bypass grafts; experts recommended that additional treatment with SAPT should be considered. In these scenarios, a consensus was achieved that the criteria defined to restore the antithrombotic regimen (Table 5) should be considered on an individual basis.

#### 3.2.3. Dual Antiplatelet Therapy Due to a Recent PCI

For patients on DAPT due to a recent PCI (<6 months), experts recommended that DAPT should be continued after discharge for all bypass procedures (Table 4). No additional antithrombotic therapy was recommended in any of these scenarios. A consensus was achieved that patients should be treated as those with no previous antithrombotic therapy (Table 2) after discontinuing cardiac DAPT.

### 3.3. Treatment Durations

Recommendations on treatment durations were not studied as a Delphi consensus. Durations were reported by the experts and the durations covering 75% of the reports were presented as ranges for each scenario in Table 2, Table 3 and Table 4.

Table 5 summarizes the criteria that experts proposed to be considered before the antithrombotic regimen is restored to the preoperative state. Consensus was achieved on four systemic criteria: “Risk of falls”, “Freedom from major bleeding”, “Freedom from clinical progression of cerebrovascular or cardiac atherosclerosis”, and “Known failure of previous antithrombotic treatment in PAOD”. Consensus was achieved on eight bypass criteria: “Bypass flow”, “Duration since surgery”, “Bypass anatomy”, “Freedom from anastomotic stenosis”, “Bypass material”, “Quality of vein graft”, “Distal run-off”, and “Pre-existence of ipsilateral arterial stents or angioplasty”. Finally, consensus was achieved on rejecting “Improvement of peripheral wound situation” and “Improvement of claudication”. No consensus was achieved on “Intraoperative bypass-flow”, “Smoking cessation”, and “Results of platelet function tests”.

## 4. Discussion

This Delphi consensus document complements the ESVS guidelines and provides expert consensus on antithrombotic therapy in 25 clinical scenarios for bypass surgery in patients with PAOD. The opinions of the experts surveyed cover a wide spectrum of clinical scenarios; many of which are not covered by the current global guidelines.

**Clinical scenario 1—**isolated PAOD: In patients without prior antithrombotic therapy, a consensus was achieved for using SAPT in above-the-knee popliteal artery bypasses regardless of the graft material used. This is in line with the recommendations of the ESVS guidelines. However, evidence from a subgroup analysis of the BOA study is often cited to support the use of an oral vitamin K antagonist after venous bypass even if the distal anastomosis is above the knee [3,13]. One explanation for this result might be a devaluation of the historical evidence from the BOA study by the expert group.

For below-the-knee popliteal artery bypasses, a consensus was only achieved on autologous and biological grafts. For this, the experts recommended DPI based on the recent findings from the VOYAGER PAD [5]. This recommendation contrasts with the one from the current guidelines about using full-dose anticoagulants for below-the-knee popliteal artery bypass grafts [10]. This is not surprising, considering that the guidelines were finalized before the results of the VOYAGER PAD were available. Recently, using DPI was incorporated into the 2022 PAOD guidelines of the Canadian Cardiovascular Society (CCS) [6], the 2021 European Society of Cardiology (ESC) consensus document on the antithrombotic therapies in aortic and peripheral arterial disease [7], and the 2019 PAOD guidelines of the European Society of Vascular Medicine (ESVM) [14].

Interestingly, no consensus was achieved on prosthetic below-the-knee popliteal artery bypass grafts. Of the experts, 56% recommended DAPT. This recommendation was mainly based on the results of the CASPAR trial that studied the below-knee bypass grafts. It only showed significant results for a non-stratified prosthetic bypass subgroup. The remaining 44% recommended DPI based on the more recent VOYAGER PAD protocol that studied a very wide range of PAOD patients [4,5]. Full-dose OAC as monotherapy or in combination with antiplatelet therapy was rejected in the first round of the Delphi process.

**Clinical scenario 2—**atrial fibrillation requiring OAC: Experts achieved a consensus that OAC should be continued after the bypass surgery if it was indicated for atrial fibrillation. For a distal prosthetic bypass, the experts recommended adding SAPT. For below-the-knee popliteal artery prosthetic or biological bypass grafts and patients with distal vein or biological bypass grafts, they recommended consideration of additional SAPT. OAC + SAPT in patients with PAOD was evaluated in the “Warfarin Antiplatelet Vascular Evaluation Trial” (WAVE trial) [15]. It showed that the combination of an oral anticoagulant and antiplatelet therapy was not more effective than antiplatelet therapy alone in preventing major cardiovascular complications but was associated with an increase in life-threatening bleeding complications.

None of the experts recommended triple therapy even after a prosthetic below-the-knee bypass. This is in line with the currently recommended treatment regimens for patients with atrial fibrillation who had undergone PCI [16]. Three large randomised clinical trials (RCTs) showed that for these patients, SAPT + DOAC had significantly fewer bleeding events than the classical triple therapy with comparable efficacy [17,18,19]. It should be noted that various DOAC regimens were used in those trials; however, the approved doses for stroke prevention were utilized. Dose reduction was performed according to the individual’s DOAC dose reduction criteria [16].

This study did not address perioperative management, including the timing of the initiation of full-dose anticoagulation (oral, subcutaneous, or intravenous) after surgery. A thorough assessment of the risk of postoperative bleeding and the risk of ischaemic events in patients with atrial fibrillation is of paramount importance. We recommend consulting the current ESC Guidelines on cardiovascular assessment and management of patients undergoing non-cardiac surgery, which cover perioperative antithrombotic management [20].

**Clinical scenario 3—**recent PCI requiring DAPT: The experts reached a consensus that all patients in this scenario should remain on DAPT regardless of the anatomy and material of the bypass. The clear stance against triple therapy has a solid rationale on the basis of mitigating bleeding events and complications [17,18,19]. It is worth noting that the efficacy of triple therapy on the patency of peripheral bypass and local complications has not been studied.

**Treatment duration:** Treatment regimen and duration should be decided on an individualised basis and reviewed regularly. The expert group showed a high degree of agreement on a proposed set of criteria that can influence the administration of antithrombotic drugs (Table 5). They rejected two of the proposed criteria: “Improvement of claudication” and “Wound situation”.

Assessing the risk-benefit profile of antithrombotic therapy in the context of peripheral bypass surgery often requires substantial clinical experience. However, several scores can support evidence-based recommendations. The HAS-BLED score, which factors arterial hypertension, abnormal renal or liver function, stroke, history of or predisposition of bleeding, labile INR, age > 65, and drugs or alcohol, is a validated score for assessing bleeding risk in the context of anticoagulation in atrial fibrillation [21]. That score was applied to a retrospective cohort of patients with anticoagulation after surgical revascularisation of the lower extremity. The results matched the increased risk of major bleeding with the increased HAS-BLED scores in that context [22]. In addition, the OAC3-PAD risk score predicts major bleeding using clinical data for symptomatic PAOD patients. The independent predictors were previous oral anticoagulation, age > 80, CLTI, congestive heart failure, chronic kidney disease, previous bleeding events, anaemia, and dementia [9]. The routine use of these scores seems reasonable, but they should be further investigated with prospective studies.

It is noteworthy that several experts recommended limiting DPI therapy after a certain time following the bypass procedure. This recommendation contradicts the recommendations of the VOYAGER-PAD trial, which recommends an unlimited prescription of DPI after lower limb revascularisation [5]. However, this could be interpreted as a recommendation to consistently reassess the indications for antithrombotic therapy, as the risk-benefit profile may change.

**Limitations:** The experts’ consensus statements represent the lowest level of evidence. They offer guidance in clinical situations not covered by the current global guidelines but must be interpreted within their limitations [11].

First, not all potentially eligible experts had been contacted and a random selection of the experts was not feasible. Further, the proportion of non-responders during the Delphi process was relatively high. However, there were no systematic differences between completers and non-completers and the self-reported experience of the expert group was very high; with a reported institutional caseload of > 840 bypasses for PAOD over the last decade. Nevertheless, the consensus achieved by this expert group might not reflect the real European expertise and opinions.

Second, Delphi studies have intrinsic limitations and have been criticised because the content is chosen by the research team, potentially introducing bias. To minimise this risk, the experts had the opportunity to comment on the statements or propose different treatment strategies and additional criteria during the process. However, not all surgical revascularisation options are covered by this survey, including multi-segment bypasses or bypasses with venous arterializations.

Third, the expert recommendations might be driven by local regulations including availability of different medications, local prices, or reimbursement systems for patients.

Finally, there are other clinical scenarios not covered by this survey. For example, the antithrombotic treatment regimen after bypass surgery in patients with pre-existing OAC due to previous pulmonary embolism or deep vein thrombosis, or treatment with DAPT due to recent infrainguinal arterial interventions. The three clinical scenarios were proposed by the interdisciplinary research group and reflect expert opinion on the most important clinical situations rather than a conclusive list.

## 5. Conclusions

This expert consensus study shows that antithrombotic therapy in patients after bypass surgery is very heterogeneous and high-quality data on the optimal antithrombotic treatment regimen is lacking. Recent RCTs have suggested the use of DPI after the revascularisation of PAOD. However, this study demonstrated that these new findings have not been widely adopted yet by patients undergoing bypass surgery. Individualised decision-making is required in these complex scenarios. The expert consensus statements provide guidance for clinical situations not covered by the current ESVS guidelines but must be interpreted within its limitations.

## Figures and Tables

**Table 1:** Consensus statements on antithrombotic therapy for patients with isolated PAOD with no intervention.

Consensus Statements	Agreement ^1^
Patients with asymptomatic peripheral artery disease (Fontaine Stage I, Rutherford Grade 0) should receive ASA.	76% R2
Patients with mild peripheral artery disease (Fontaine Stage IIa, Rutherford Grade 1) should receive ASA. ^2^	77% R2

^1^ Consensus was achieved if ≥75% proposed the same treatment regimen in rounds 1 to 4 (R1–R4). ^2^ ASA = acetylsalicylic acid.

**Table 2:** Consensus statements for patients with no previous antithrombotic therapy.

Consensus Statements	Agreement ^1^
Patients with above-the-knee popliteal artery autologous bypass should permanently receive single antiplatelet therapy.	82% R3
Patients with above-the-knee popliteal artery prosthetic bypass should permanently receive single antiplatelet therapy.	85% R3
Patients with above-the-knee popliteal artery biological bypass should permanently receive single antiplatelet therapy.	82% R3
Patients with below-the-knee popliteal artery autologous bypass should permanently receive single antiplatelet therapy. Dual pathway inhibition for 6 months up to permanent treatment should be considered.	93% R4
Patients with below-the-knee popliteal artery prosthetic bypass. 56% dual antiplatelet therapy vs. 44% Dual pathway inhibition	no consensus
Patients with below-the-knee popliteal artery biological bypass should permanently receive single antiplatelet therapy. Dual pathway inhibition for 12 months up to permanent treatment should be considered.	85% R4
Patients with distal autologous bypass should permanently receive single antiplatelet therapy. Additional low-dose oral anticoagulation for 6 months up to permanent treatment should be considered.	76% R3
Patients with distal prosthetic bypass. 67% dual pathway inhibition vs. 33% single antiplatelet therapy + oral anti-coagulation	no consensus
Patients with distal biological bypass should permanently receive single antiplatelet therapy. Additional low-dose or full-dose oral anticoagulation for 6 months up to permanent treatment should be considered.	76% R4

^1^ Consensus was achieved if ≥75% proposed the same treatment regimen in rounds 1 to 4 (R1–R4). For dissensus, the top two most frequent answers are presented.

**Table 3:** Consensus statements for patients with previous OAC due to atrial fibrillation.

Consensus Statements	Agreement ^1^
Patients with above-the-knee popliteal artery autologous bypass should permanently receive oral anticoagulation.	100% R3
Patients with above-the-knee popliteal artery prosthetic bypass should permanently receive oral anticoagulation.	96% R3
Patients with above-the-knee popliteal artery biological bypass should permanently receive oral anticoagulation.	96% R3
Patients with below-the-knee popliteal artery autologous bypass should permanently receive oral anticoagulation.	78% R3
Patients with below-the-knee popliteal artery prosthetic bypass should permanently receive oral anticoagulation. Additional single antiplatelet therapy for 3 months up to permanent therapy should be considered.	84% R4
Patients with below-the-knee popliteal artery biological bypass should permanently receive oral anticoagulation. Additional single antiplatelet therapy for 3 months up to permanent therapy should be considered.	84% R4
Patients with distal autologous bypass should permanently receive oral anticoagulation. Additional single antiplatelet therapy for 6 months up to permanent therapy should be considered.	99% R4
Patients with distal prosthetic bypass should permanently receive oral anti-coagulation + additional single antiplatelet therapy for 3 months up to permanent therapy.	78% R3
Patients with distal biological bypass should permanently receive oral anticoagulation. Additional single antiplatelet therapy for 3 months up to permanent therapy should be considered.	92% R4

^1^ Consensus was achieved if ≥75% proposed the same treatment regimen in rounds 1 to 4 (R1–R4).

**Table 4:** Consensus statements for patients with previous DAPT due to coronary intervention.

Consensus Statements	Agreement ^1^
Patients with above-the-knee popliteal artery autologous bypass should continue dual antiplatelet therapy.^2^	82% R1
Patients with above-the-knee popliteal artery prosthetic bypass should continue dual antiplatelet therapy. ^2^	79% R1
Patients with above-the-knee popliteal artery biological bypass should continue dual antiplatelet therapy. ^2^	81% R1
Patients with below-the-knee popliteal artery autologous bypass should continue dual antiplatelet therapy. ^2^	80% R2
Patients with below-the-knee popliteal artery prosthetic bypass should continue dual antiplatelet therapy. ^2^	90% R2
Patients with below-the-knee popliteal artery biological bypass should continue dual antiplatelet therapy. ^2^	83% R2
Patients with distal autologous bypass should continue dual antiplatelet therapy. ^2^	79% R2
Patients with distal prosthetic bypass should continue dual antiplatelet therapy. ^2^	93% R3
Patients with distal biological bypass should continue dual antiplatelet therapy. ^2^	76% R3

^1^ Consensus was achieved if ≥75% proposed the same treatment regimen in rounds 1 to 4 (R1–R4). ^2^ For all scenarios, consensus was reached that patients should be treated according to the “no prior antithrombotic therapy” scenario at the time of dual antiplatelet therapy discontinuation (see Table 2).

**Table 5:** Criteria to restore antithrombotic therapy regimen.

Consensus Statements	Agreement ^1^
Systemic criteria:	
Risk for falls	93% R2
Known failure of previous antithrombotic treatment in PAOD ^2^	83% R2
Freedom from clinical progression of cerebrovascular or cardiac atherosclerosis	78% R3
Freedom from major bleeding events	77% R1
Patients with below-the-knee popliteal artery biological bypass should continue dual antiplatelet therapy.	83% R2
Bypass criteria:	
Bypass flow (hemodynamic on colour-duplex sonography)	89% R3
Bypass anatomy (i.e., above-the-knee popliteal artery vs. below-the-knee popliteal artery vs. distal)	87% R2
Duration since bypass surgery	87% R2
Freedom from anastomotic stenoses	85% R3
Quality of vein graft	78% R3
Distal run-off (i.e., patency and flow of arteries distal to the bypass)	77% R1
Pre-existing/concomitant ipsilateral arterial stent or angioplasty	77% R2
Bypass material (i.e., prosthetic vs. autologous vs. biological)	77% R2
Rejected criteria:	Disagreement
Improvement of claudication (i.e., pain-free walking distance)	89% R3
Improvement of peripheral wound situation	83% R2
Criteria with no consensus after three rounds:	Agreement
Intraoperative bypass-flow	56% R3
Smoking cessation/persistent smoking after bypass	56% R3
Results of platelet function tests	59% R3

^1^ Consensus was achieved if ≥75% strongly agreed or disagreed in rounds 1 to 3 (R1–R3). ^2^ PAOD = peripheral arterial occlusive disease.

## Data Availability

The data presented in this study are openly available in FigShare at https://figshare.com/articles/dataset/Data_Patent_Delphi_xlsx/22215658 (accessed on 28th April 2023).

## Supplementary Materials

The following supporting information can be downloaded at: https://www.mdpi.com/article/10.3390/jcm12093223/s1, Table S1: Expert group and institutional experience.

## References

[1] Farber A., Menard M.T., Conte M.S., Kaufman J.A., Powell R.J., Choudhry N.K., Hamza T.H., Assmann S.F., Creager M.A., Cziraky M.J. (2022). Surgery or Endovascular Therapy for Chronic Limb-Threatening Ischmia. N. Engl. J. Med..

[2] Antiplatelet Trialists’ Collaboration (1994). Collaborative overview of randomised trials of antiplatelet therapy--II: Maintenance of vascular graft or arterial patency by antiplatelet therapy. BMJ.

[3] (2000). Efficacy of oral anticoagulants compared with aspirin after infrainguinal bypass surgery (The Dutch Bypass Oral Anticoagulants or Aspirin Study): A randomised trial. Lancet.

[4] Belch J., Dormandy J., Biasi G., Cairols M., Diehm C., Eikelboom B. (2010). Results of the randomized, placebo-controlled clopidogrel and acetylsalicylic acid in bypass surgery for peripheral arterial disease (CASPAR) trial. J. Vasc. Surg..

[5] Bonaca M.P., Bauersachs R.M., Anand S.S., Debus E.S., Nehler M.R., Patel M.R., Fanelli F., Capell W.H., Diao L., Jaeger N. (2020). Rivaroxaban in Peripheral Artery Disease after Revascularization. N. Engl. J. Med..

[6] Abramson B.L., Al-Omran M., Anand S.S., Albalawi Z., Coutinho T., de Mestral C., Dubois L., Gill H.L., Greco E., Guzman R. (2022). Canadian Cardiovascular Society 2022 Guidelines for Peripheral Arterial Disease. Can. J. Cardiol..

[7] Aboyans V., Bauersachs R., Mazzolai L., Brodmann M., Palomares J.F.R., Debus S., Collet J.-P., Drexel H., Espinola-Klein C., Lewis B.S. (2021). Antithrombotic therapies in aortic and peripheral arterial diseases in 2021: A consensus document from the ESC working group on aorta and peripheral vascular diseases, the ESC working group on thrombosis, and the ESC working group on cardiovascular pharma. Eur. Heart J..

[8] De Carlo M., Schlager O., Mazzolai L., Brodmann M., Espinola-Klein C., Staub D., Aboyans V., Sillesen H., Debus S., Venermo M. (2022). Antithrombotic therapy following revascularization for chronic limb-threatening ischaemia: A European survey from the ESC Working Group on Aorta and Peripheral Vascular Diseases. Eur. Heart. J. Cardiovasc. Pharmacother..

[9] Behrendt C.-A., Kreutzburg T., Nordanstig J., Twine C.P., Marschall U., Kakkos S., Aboyans V., Peters F. (2022). The OAC(3)-PAD Risk Score Predicts Major Bleeding Events one Year after Hospitalisation for Peripheral Artery Disease. Eur. J. Vasc. Endovasc. Surg..

[10] Gage B.F., Van Walraven C., Pearce L., Hart R.G., Koudstaal P.J., Boode B.S.P., Petersen P. (2004). Selecting patients with atrial fibrillation for anticoagulation: Stroke risk stratification in patients taking aspirin. Circulation.

[11] Conte M.S., Bradbury A.W., Kolh P., White J.V., Dick F., Fitridge R., Mills J.L., Ricco J.-B., Suresh K.R., Murad M.H. (2019). Global Vascular Guidelines on the Management of Chronic Limb-Threatening Ischemia. Eur. J. Vasc. Endovasc. Surg..

[12] Anand S.S., Bosch J., Eikelboom J.W., Connolly S.J., Diaz R., Widimsky P., Aboyans V., Alings M., Kakkar A.K., Keltai K. (2018). Rivaroxaban with or without aspirin in patients with stable peripheral or carotid artery disease: An international, randomised, double-blind, placebo-controlled trial. Lancet.

[13] Aboyans V., Ricco J.B., Bartelink M.L., Björck M., Brodmann M., Cohner T., Collet J.P., Czerny M., De Carlo M., Debus S. (2018). Editor’s Choice—2017 ESC Guidelines on the Diagnosis and Treatment of Peripheral Arterial Diseases, in collaboration with the European Society for Vascular Surgery (ESVS). Eur. J. Vasc. Endovasc. Surg..

[14] Frank U., Nikol S., Belch J., Boc V., Brodmann M., Carpentier P.H., Chraim A., Canning C., Dimakakos E., Gottsäter A. (2019). ESVM Guideline on peripheral arterial disease. Vasa.

[15] Anand S., Yusuf S., Xie C., Pogue J., Eikelboom J., Budaj A., Warfarin Antiplatelet Vascular Evaluation Trial Investigators (2007). Oral anticoagulant and antiplatelet therapy and peripheral arterial disease. N. Engl. J. Med..

[16] Steffel J., Collins R., Antz M., Cornu P., Desteghe L., Haeusler K.G., Oldgren J., Reinecke H., Roldan-Schilling V., Rowell N. (2021). 2021 European Heart Rhythm Association Practical Guide on the Use of Non-Vitamin K Antagonist Oral Anticoagulants in Patients with Atrial Fibrillation. Europace.

[17] Lopes R.D., Heizer G., Aronson R., Vora A.N., Massaro T., Mehran R., Goodman S.G., Windecker S., Darius H., Li J. (2019). Antithrombotic Therapy after Acute Coronary Syndrome or PCI in Atrial Fibrillation. N. Engl. J. Med..

[18] Gibson C.M., Mehran R., Bode C., Halperin J., Verheugt F.W., Wildgoose P., Birmingham M., Ianus J., Burton P., van Eickels M. (2016). Prevention of Bleeding in Patients with Atrial Fibrillation Undergoing PCI. N. Engl. J. Med..

[19] Cannon C.P., Bhatt D.L., Oldgren J., Lip G.Y., Ellis S.G., Kimura T., Maeng M., Merkely B., Zeymer U., Gropper S. (2017). Dual Antithrombotic Therapy with Dabigatran after PCI in Atrial Fibrillation. N. Engl. J. Med..

[20] Halvorsen S., Mehilli J., Cassese S., Hall T.S., Abdelhamid M., Barbato E., De Hert S., de Laval I., Geisler T., Hinterbuchner L. (2022). 2022 ESC Guidelines on cardiovascular assessment and management of patients undergoing non-cardiac surgery. Eur. Heart J..

[21] Pisters R., Lane D.A., Nieuwlaat R., De Vos C.B., Crijns H.J., Lip G.Y. (2010). A novel user-friendly score (HAS-BLED) to assess 1-year risk of major bleeding in patients with atrial fibrillation: The Euro Heart Survey. Chest.

[22] Freixo C., Ferreira V., Gonçalves J., Teixeira G., Antunes I., Veiga C., Mendes D., Veterano C., Martins J., Almeida R. (2019). HAS-BLED Score Predicts the Risk of Major Bleeding in Chronic Anticoagulation after Lower Limb Surgical Revascularization. Ann. Vasc. Surg..

