# Supplementary material for: Preventing Lower Limb Graft Thrombosis after Infrainguinal Arterial Bypass Surgery with Antithrombotic Agents (PATENT Study): An International Expert Based Delphi Consensus
Source: J Clin Med. 2023 Apr 30;12(9):3223. doi: 10.3390/jcm12093223 (PMC10178874; doi:10.3390/jcm12093223)
Supplement: Supplementary file 1 [file jcm-12-03223-s001.zip › jcm-2294202-supplementary.pdf]

Supplementary Table S1. Expert group and institutional experience

Characteristics of experts and their institutions	Total Round 1 n=39	Total Round 1-4 n=28
Country of residence:		
Germany	7 (18%)	4 (14%)
Austria	5 (13%)	3 (11%)
Switzerland	5 (13%)	4 (14%)
Sweden	4 (10%)	3 (11%)
Greece	2 (5%)	2 (7%)
Italy	2 (5%)	2 (7%)
Netherlands	2 (5%)	2 (7%)
United Kingdom	1 (3%)	1 (4%)
Belgium	1 (3%)	1 (4%)
Croatia	1 (3%)	1 (4%)
Malta	2 (5%)	1 (4%)
France	2 (5%)	1 (4%)
Hungary	1 (3%)	1 (4%)
Romania	1 (3%)	1 (4%)
Spain	1 (3%)	1 (4%)
Denmark	1 (3%)	-
Finland	1 (3%)	-
Medical speciality:		
Vascular Surgery	35 (90%)	25 (89%)
Angiology	3 (8%)	2 (7%)
Interventional Cardiology	1 (3%)	1 (4%)
**Institutional experience by type of procedure (caseload / year)**		
Above-the-knee popliteal artery bypass, median (Q1 to Q3)	29 (19 to 50)	26 (19 to 52)
Below-the-knee popliteal artery bypass, median (Q1 to Q3)	50 (29 to 71)	38 (24 to 61)
Distal bypass, median (Q1 to Q3)	20 (10 to 26)	20 (10 to 24)
**Preferred graft material***		
Above-the-knee popliteal artery	1. Autologous (31)<br>2. Prosthetic (8)<br>3. Biological (0)	1. Autologous (21)<br>2. Prosthetic (7)<br>3. Biological (0)
Below-the-knee popliteal artery	1. Autologous (35)<br>2. Prosthetic (2)<br>3. Biological (1)	1. Autologous (26)<br>2. Prosthetic (2)<br>3. Biological (0)
Distal	1. Autologous (38)<br>2. Prosthetic (1)<br>3. Biological (0)	1. Autologous (28)<br>2. Prosthetic (0)<br>3. Biological (0)

Data are presented as n (%) or median (Q1 = quartile 1 to Q3 = quartile 3) for institutional caseload.
* Preferred graft material presented as ranks (1. - 3.) with number of votes in brackets. One expert did not choose a preferred graft material for below-the-knee popliteal artery bypass.
